# Detection of Polyphonic Alarm Sounds From Medical Devices Using Frequency-Enhanced Deep Learning: Simulation Study

**DOI:** 10.2196/35987

**Published:** 2025-11-12

**Authors:** Kazumasa Kishimoto, Tadamasa Takemura, Osamu Sugiyama, Ryosuke Kojima, Masahiro Yakami, Goshiro Yamamoto, Tomohiro Kuroda

**Affiliations:** 1 Graduate School of Informatics, Kyoto University, 54 Kawara-cho, Shogoin, Sakyo-ku, Kyoto, 591-8022, Japan, 81 75-366-7701; 2 Graduate School of Medicine, Kyoto University, Kyoto, Japan; 3 Kyoto University Hospital, Kyoto, Japan; 4 Graduate School of Information Science, University of Hyogo, Kobe, Japan; 5 Department of Information Science, Kindai University, Osaka, Japan

**Keywords:** sound event detection, deep learning, alarm sound, polyphonic sound, notifications

## Abstract

**Background:**

Although an increasing number of bedside medical devices are equipped with wireless connections for reliable notifications, many nonnetworked devices remain effective at detecting abnormal patient conditions and alerting medical staff through auditory alarms. Staff members, however, can miss these notifications, especially when in distant areas or other private rooms. In contrast, the signal-to-noise ratio of alarm systems for medical devices in the neonatal intensive care unit is 0 dB or higher. A feasible system for automatic sound identification with high accuracy is needed to prevent alarm sounds from being missed by the staff.

**Objective:**

The purpose of this study was to design a method for classifying multiple alarm sounds collected with a monaural microphone in a noisy environment.

**Methods:**

Features of 7 alarm sounds were extracted using a mel filter bank and incorporated into a classifier using convolutional and recurrent neural networks. To estimate its clinical usefulness, the classifier was evaluated with mixtures of up to 7 alarm sounds and hospital ward noise.

**Results:**

The proposed convolutional recurrent neural network model was evaluated using a simulation dataset of 7 alarm sounds mixed with hospital ward noise. At a signal-to-noise ratio of 0 dB, the best-performing model (convolutional neural network 3+bidirectional gate recurrent unit) achieved an event-based *F*_1_-score of 0.967, with a precision of 0.944 and a recall of 0.991. When the venous foot pump class was excluded, the classwise recall of the classifier ranged from 0.990 to 1.000.

**Conclusions:**

The proposed classifier was found to be highly accurate in detecting alarm sounds. Although the performance of the proposed classifier in a clinical environment can be improved, the classifier could be incorporated into an alarm sound detection system. The classifier, combined with network connectivity, could improve the notification of abnormal status detected by unconnected medical devices.

## Introduction

### Background

While an increasing number of bedside medical devices, such as syringe pumps, have wireless connections that enable reliable data transmission to hospital information systems, many nonnetworked devices are still used in general hospital wards. Although dongles connected to the external output terminal of these devices may allow wireless connections [1,2], most devices are not equipped with external output terminals. Instead, these devices use auditory alert signals (alarm sounds) to notify medical staff of abnormal conditions. Medical staff members may not hear these alarms, especially when they are in distant areas or other private rooms. Between 2010 and 2015, the Japan Council for Quality Health Care reported 173 accidents and other incidents, including 23 cases of unnoticed alarms [3]. The report included the following comments about environmental factors:

There are many blind spots due to the facility’s structure.

When I entered a patient’s room, I could not hear alarm sounds from another room.

The alarm sound did not reach the staff because the room was far from the nurse station.

The staff could not hear the alarm in the farthest private room in the ICU.

The recording room was structured so that the alarm sound could not be heard.

These findings indicate the need for reliable alarm notification to ensure patient safety. Alarm sounds emitted by medical devices are regulated by the International Organization for Standardization and the International Electrotechnical Commission (ISO/IEC) 60601-1-8. This standard specifies the melodies and lengths of alarm sounds to reduce the risk of misunderstanding, confusion, and omission of alarm sounds from various medical devices, even when these sounds overlap and reverberate. This standard prescribes that the sounds should be organized based on the priority of corresponding abnormal situations, with alarm sounds for different situations varying in melody and length. Thus, sound event detection (SED) is expected to identify every kind of abnormal situation detected by medical devices [4-8]. The standard also defines visual alarm signals, but monitoring the signals of multiple devices with cameras is not feasible without blind spots.

As SED can be implemented using a single (monaural) microphone, it was selected as the approach to detect alarm status. Clinical application of SED requires robustness against noise because environmental noise in hospital wards is generally substantial. SEDs with deep learning have been found to be sufficiently robust against noise [9,10].

This study proposes a deep learning–based method for classifying patient abnormalities detected by medical devices with polyphonic alarm sounds collected with a monaural microphone. The ability of the classifier to identify abnormal states of these devices was evaluated using simulation datasets of their alarm sounds superimposed on hospital ward noise (HWN). Therefore, the objective of this study was to design and evaluate a convolutional recurrent neural network (CRNN) for accurately detecting and classifying multiple, overlapping alarm sounds from medical devices in a simulated noisy hospital environment. We hypothesized that a hybrid CRNN model could achieve high performance suitable for clinical application by effectively capturing both the spectral and temporal features of the alarm sounds.

### Related Works

Accurate transmission of alarms from unconnected medical devices requires precise recognition of visual and auditory alarms. Several studies have reported high accuracy in detecting simultaneous alarm sounds mixed with a substantial level of environmental noise [4-8]. For SED, deep learning is more robust against noise than conventional methods [9,10]. On the basis of these studies, an edge device was placed at the patient’s bedside to monitor abnormal conditions detected by multiple medical devices with individual alarm sounds. In our previous study, the classifier had *F*_1_-scores of 0.727 at signal-to-noise ratios (SNRs) of 0 dB and applied a convolutional neural network (CNN) [11]. To our knowledge, no previous study has used a deep learning–based recurrent neural network (RNN) to detect polyphonic alarm sounds emitted by medical devices. Recent advances in attention-based architectures, such as the audio spectrogram transformer and conformer, have demonstrated strong performance in general SED tasks [12,13]. These models use self-attention mechanisms to capture long-range dependencies, potentially enhancing robustness in noisy environments. Exploring such transformer-based approaches for clinical alarm sound detection remains an important direction for future research.

## Methods

### Overview

The first approach to SED combines a Gaussian mixture model with a hidden Markov model, using features such as the mel-frequency cepstral coefficient from traditional methods of speech recognition [14,15].

Other approaches include the separation of sound sources by matching them using a template extracted from the input sound. This can be achieved by sound source separation techniques, such as nonnegative matrix factorization. Nonnegative matrix factorization monitors a single signal to create a basis matrix and identifies the separated sounds [16-18].

Recent approaches based on neural networks have significantly improved the performance of SED. One approach consists of SED of real-life sounds with feedforward neural networks based on a multilayer perceptron trained in a spectrum of mixed sounds [6]. An RNN with the ability to remember past states can process sequential information of the acoustic signal. RNNs with bidirectional long short-term memory have achieved excellent results in complex audio detection such as speech recognition and polyphonic piano note transcription [19-21].

Furthermore, CNNs commonly used in image recognition can robustly predict sounds with its filter shifted by both time and frequency axes [22]. However, long-term prediction remains difficult due to the limited width of the time window [9]. Therefore, although alarm sounds consist of relatively simple tones, it is necessary to predict not only the frequency axis but also the time axis to inform the priority with a pattern. Application of an RNN to polyphonic SED enabled long-term prediction by integrating the information over the time window. This study combines the strengths of both CNN and RNN to benefit from both approaches. A similar approach has shown excellent performance in automatic speech recognition [23,24].

Therefore, this approach was expected to achieve sufficient performance for potential clinical application in alarm sound recognition.

### Deep Alarm Sound Detection

#### Experiment Overview

**Figure 1.**
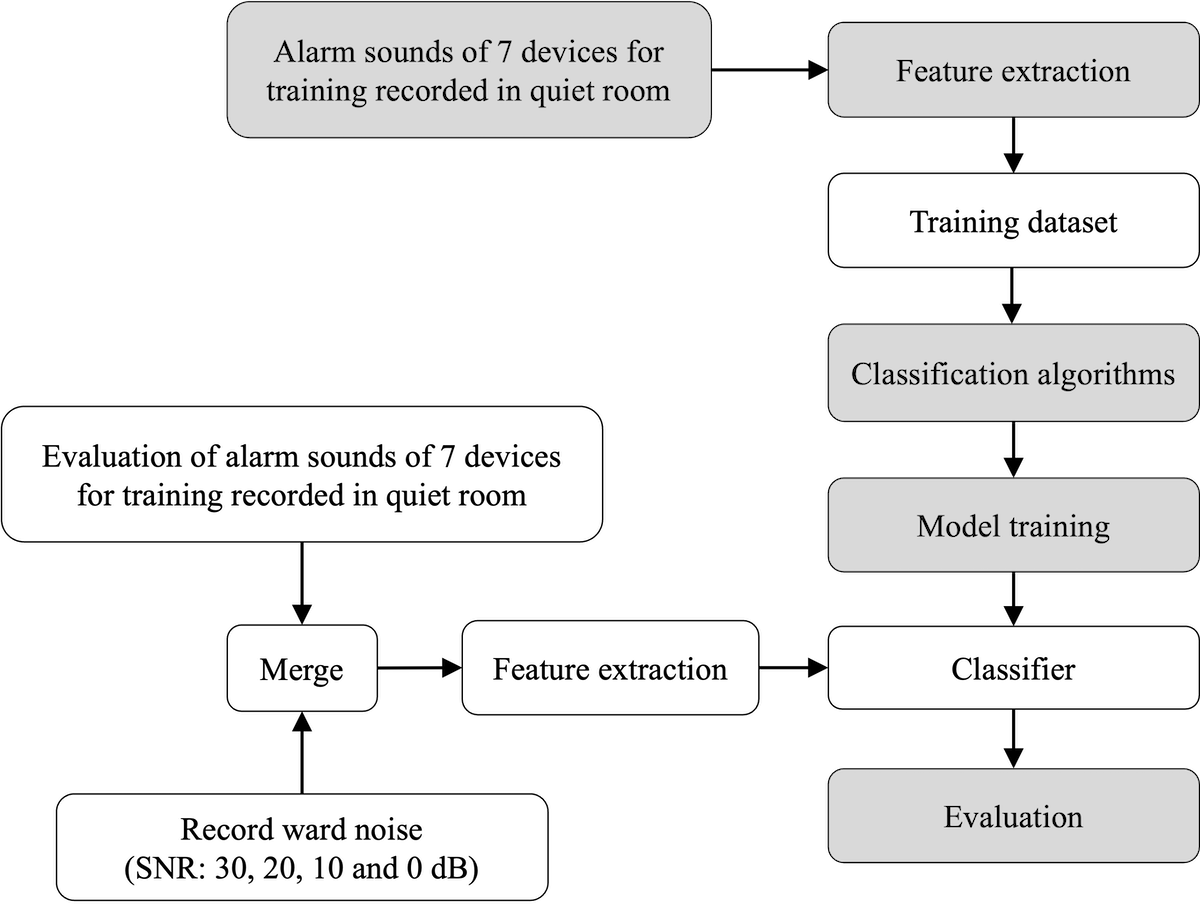
Overview of the experiment. SNR: signal-to-noise ratio.

The proposed polyphonic alarm sound detection consisted of feature extraction, classification algorithms, and model training (Figure 1). A mel filter bank (MFB) was used to extract the features of alarm sounds, and CNN and RNN were applied to the classifier. As deep learning requires substantial training data, a large amount of acoustic data were recorded in a quiet room. The recorded data were mixed with pseudo noise before being used for training. This approach aimed to maximize the generalizability of the classifier for expected use in a noisy environment. The feature extraction step extracted data to be input to the classifier from the collected alarm sounds. The classification algorithms were designed to use deep learning models for classifiers. The model training augmented the data to create a robust model.

#### Feature Extraction

The features of polyphonic alarm sounds of bedside medical devices were extracted. Many acoustic classifiers use log mel spectrogram multiplied spectra and MFB based on the characteristics underlying human frequency perception [25]. The acoustic data were transformed to a power spectrogram with the short-time Fourier transform of the Hamming window, which had a window size of 1024 and a hop length of 512. The inner product on the power spectrogram was calculated with MFB, and its logarithms in 40 dimensions were calculated.

#### Classification Algorithms

**Figure 2.**
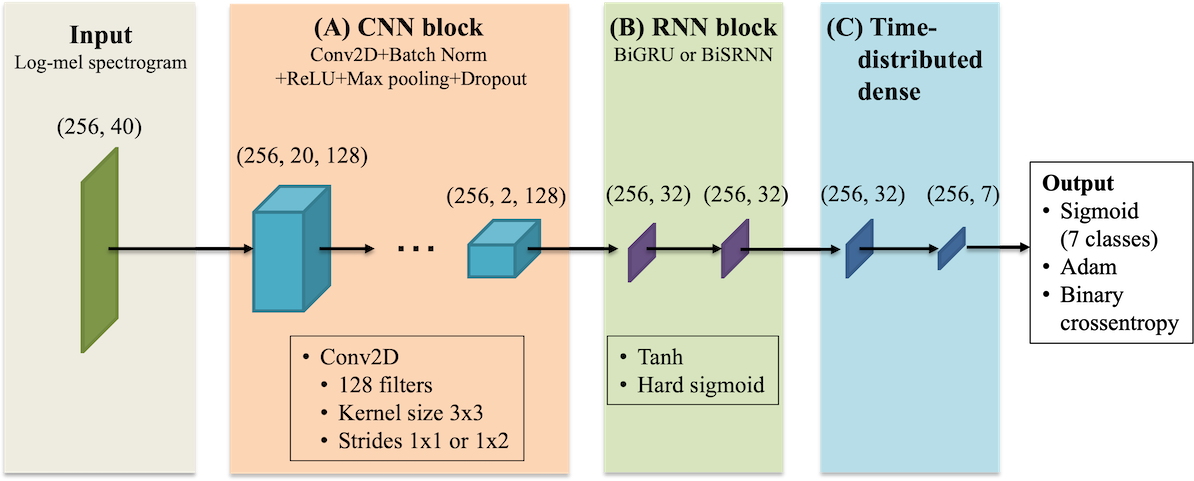
Architecture of the proposed convolutional recurrent neural network. The architecture consists of three main components: (1) convolutional neural network (CNN) block (convolutional layers with batch normalization, rectified linear unit [ReLU] activation, max pooling, and dropout), (2) recurrent neural network (RNN) block (bidirectional gate recurrent unit [BiGRU] or bidirectional simple recurrent neural network [BiSRNN] layers), and (3) time-distributed dense with sigmoid activation for classification.

The CRNN, a combination of CNN and RNN, was used for the classification model (Figure 2) [26]. This system used 256×40 pixels of the log mel spectrogram as a feature. A CNN block in the proposed model consisted of a convolutional layer with 128 filters, batch normalization, the activation function of the rectified linear unit, and a dropout of 50% [27]. Using the size reduction method, the max pooling layer was applied to the frequency axis. In image recognition, replacing the pooling layer with a CNN stride 2 has been reported to improve its performance by reducing the calculation cost [28]. In this study, the size of the stride to the frequency axis was reduced to 2, and the pooling layer was excluded because the classification target was 1 frame of the sound.

The RNN block consisted of a bidirectional simple RNN (BiSRNN) or bidirectional gated recurrent unit (BiGRU). The input was set to 32 units, the activation function to tanh, the recurrent activation function to the hard sigmoid, and the dropout of each layer to 50%.

Finally, the activation function of the fully connected layer was set to sigmoid, the optimization function used was Adam [29], and the loss function was set to binary cross-entropy. Each of the 7 sound event classes had output values in the range of [0, 1] [5].

Table 1 shows the details of the proposed model. CNN3+BiSRNN was used as the baseline model. The environment was built with Python programming language (version 3.7.11; Python Software Foundation), using Keras 2.3.1 for the deep learning library (TensorFlow 2.0.0 for the back end) and Librosa 0.8.1 for the acoustic analysis module. The experimental code is available online[30].

**Table 1.** Details of the proposed model.

Model name	CNN^a^ block				RNN^c^ block	
	Layer	Number of layers	Stride	Max pooling^b^	Layer	Number of layers
CNN3+BiSRNN^d^ (baseline)	Conv2D^e^	3	1×1	5, 2, 2	BiSRNN	2
CNN3+BiGRU^f^	Conv2D	3	1×1	5, 2, 2	BiGRU	2
CNN4+BiGRU	Conv2D	4	1×1	2, 2, 2, 2	BiGRU	2
ALL-CNN4+BiGRU^g^	Conv2D	4	1×2	—^h^	BiGRU	2

a CNN: convolutional neural network.

b The figures denote frequency axis. Time axis=1 (ie,1×5=5).

c RNN: recurrent neural network.

d BiSRNN: bidirectional simple recurrent neural network.

e Conv2D: two-dimensional convolution.

f BiGRU: bidirectional gated recurrent unit.

g ALL-CNN: customizing stride without max pooling.

h Not applicable.

#### Model Training

Data augmentation was applied to the training dataset to prevent overfitting of the classifier and to provide robust performance in simulation experiments [31]. The 7 alarm sounds were superimposed on white noise at SNRs ranging from 30 to 0 dB in 1 dB steps. In addition, SpecAugment was applied to the generator, with 1 random mask added for each frequency and time axis, followed by performance of 5 steps per minibatch [32].

These steps produced a trained model using 5-fold cross-validation over 150 training epochs and confirmed that the learning curve showed no signs of overfitting.

### Evaluation

#### Data Collection

**Figure 3.**
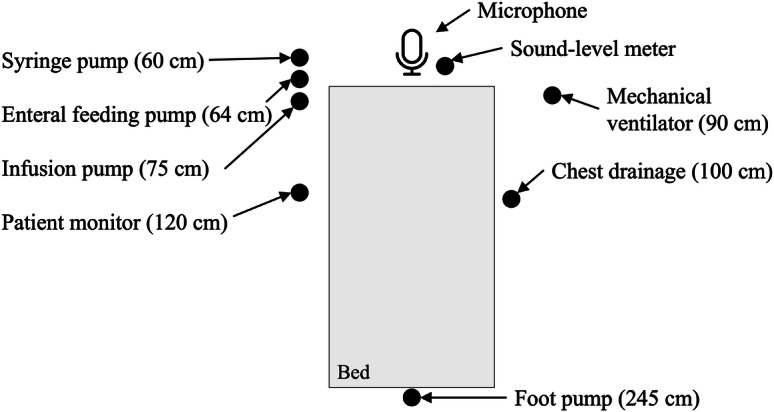
Recording environment. Numbers in parentheses represent the distance between the microphone and each device.

The medical devices selected included those frequently used for ventilator-equipped patients who require many medical devices in the general ward of the hospital. Alarm sounds to be identified included pulse sounds from a syringe pump (SP), enteral feeding pump (ENP), and venous foot pump (VFP) device as well as burst sounds from an infusion pump (IP), chest drainage (CD), patient monitor (PM), and the mechanical ventilator. The alarm sounds of each device were recorded using a monaural microphone placed at the head of a bed in a quiet private room in the hospital. The distance between the sound source and the microphone was the same as in a typical bedside setting (Figure 3). The sound pressure level was recorded simultaneously. The recording has a different number of active sound events superimposed on each frame. Therefore, the frame has various polyphony levels. The distribution of polyphony levels when recording the alarm sounds for the 7 devices is shown in Table 2. Audacity was used for labeling, extracting fundamental frequencies, and performing spectral analysis for annotation. Table 3 shows the detailed characteristics of the recorded alarm sounds.

**Table 2.** Level of each sound relative to the total level of polyphonic sound.

Polyphony level	Data mount, n (%)
0	0 (0)
1	3655 (5.7)
2	15,011 (23.6)
3	22,547 (35.4)
4	15,911 (25)
5	5280 (8.3)
6	1230 (1.9)
7	64 (0.1)

**Table 3.** Detailed characteristics of the sounds of each alarm.

Source device	Model (manufacturer)	Peak frequencies (Hz)^a^	Signal duration (seconds)	Silence duration (seconds)	Overall duration (seconds)
Infusion pump	OT-818G (JMS)	[(856,856,856)-(856,856)] 2 times	3.34	2.99	6.33
Syringe pump	TE-351 (Terumo)	4001	0.26	0.20	0.46
Enteral feeding pump	APPLIX Smart (Fresenius Kabi)	4097	0.80	0.80	1.60
Venous foot pump	SCD700 (Coviden)	2108	0.20	1.30	1.50
Chest drainage	THOPAZ (Medela)	2632	1.07	8.95	10.02
Patient monitor	PVM-4761 (NihonKoden)	[(783,994,1181)‐(1181,1569)] 2 times^b^	2.93	4.09	7.02
Mechanical ventilator	C1 (Hamilton)	491 828 662^c^	1.10	4.98	6.08

a Fundamental frequencies analyzed by Audacity. Harmonics are excluded.

b High-priority alarm sound.

c Middle-priority alarm sound.

#### Simulation Dataset

The robustness of the classifier was evaluated using a simulation dataset of alarm sounds added to HWN (which comprised conversations, footsteps, closet opening and closing sounds, intraoral suction, and ventilator exhalation sounds but not alarm sounds from other medical devices) at different SNRs (Figure 1). For the simulation dataset, the alarm sounds were recorded separately from those in the Data Collection section, using the same recording protocol (quiet private room, identical microphone placement, and device settings). During recording, all 7 devices were set to repeatedly emit alarms, sometimes overlapping due to differences in their alarm durations. Multiple alarms sometimes sounded simultaneously because of differences in the duration times of each alarm sound. Table 4 shows the maximum sound pressure of each sound source. In contrast to the training dataset—where alarm sounds were superimposed on white noise at SNRs ranging from 30 to 0 dB in 1 dB steps—the simulation dataset used for evaluation was created by superimposing the recorded HWN on alarm sounds at 4 SNR settings (30, 20, 10, and 0 dB) to reproduce realistic clinical environments. As shown in this figure, the VFP alarm exhibited the lowest sound pressure level among the devices, whereas other devices, such as SP and ENP, had relatively higher levels.

**Table 4.** Maximum sound pressure levels of each of the devices in the recording simulation dataset.

Source device	Sound level (dB)
Infusion pump	71.9
Syringe pump	77.7
Enteral feeding pump	64.4
Venous toot pump	61.2
Chest drainage	73.7
Patient monitor	62.1
Mechanical ventilator	79.6
Hospital ward noise	63.4

#### Performance Metrics

Model performance was evaluated using 5-fold cross-validation. No formal statistical significance tests were conducted, as the primary objective was descriptive benchmarking rather than hypothesis testing. Evaluation was performed using the sed_eval module [7,33]. The classifier outputs each predicted value of the 7 devices. The predicted values were dichotomized based on a cutoff value of 0.5. The onset and offset times input into the sed_eval module were calculated from the change points of the output results. Segment-based metrics are an index that determines whether the reference and the output match for each set time resolution (second). Therefore, the time resolution was set to 0.1 seconds, half of the shortest alarm duration time among the 7 devices. The event-based metrics index evaluated the timing of onset and offset from the set collar (seconds). The collar was set to 2.0 seconds based on the response of the notification system. Event-based metrics evaluation considers the actual operation and is more stringent than segment-based evaluation. Comparisons were evaluated using overall metrics (microaverage) and class-wise metrics.

For clinical applicability, inaccurate information is unacceptable, as it puts patients at risk. Therefore, the requirement for clinical application was set at an *F*_1_-score value of 0.900 or higher for event-based overall metrics and an *F* value of 0.950 or higher for class-wise recall metrics.

Finally, the predicted results were visualized using the sed_vis module, and the spectrogram and classification results were examined [34].

### Ethical Considerations

This study did not involve human participants or animal experiments. The recordings contained no speech, personal identifiers, or patient-related information. Therefore, ethics approval was not required in accordance with institutional and international research ethics policies.

## Results

Table 5 summarizes the overall performance metrics at an SNR of 0 dB. The 679-second simulation dataset consisted of 63,649 frames, of which 63,488 frames were evaluated using 256-frame input windows. Among the segment-based metrics, CNN4+BiGRU achieved the highest *F*_1_-score, followed by CNN3+BiGRU. Conversely, for event-based metrics, CNN3+BiGRU outperformed all other models, with CNN4+BiGRU ranking second. Regarding recall, event-based metrics showed values of 0.950 or higher for all BiGRU-based models, whereas segment-based metrics yielded recall values below 0.950 for all models.

**Table 5.** Overall metrics (microaverage) at a signal-to-noise ratio of 0 dB across 5-fold cross-validation. The numbers in italics represent the optimal value for the proposed models.

	Segment-based metrics, mean (SD)			Event-based metrics, mean (SD)		
	*F*_1_-score	Precision	Recall	*F*_1_-score	Precision	Recall
CNN3^a^+BiSRNN^b^ (baseline)	0.832 (0.007)	0.822 (0.009)	0.841 (0.011)	0.921 (0.003)	0.874 (0.009)	0.973 (0.008)
CNN3+BiGRU^c^	0.868 (0.008)	0.845 (0.006)	0.894 (0.012)	*0.967 (0.011)*	*0.944 (0.014)*	*0.991 (0.011)*
CNN4+BiGRU	*0.873 (0.004)*	*0.856 (0.003)*	0.890 (0.008)	0.965 (0.008)	0.942 (0.009)	0.989 (0.008)
ALL-CNN4+BiGRU	0.867 (0.010)	0.839 (0.010)	*0.896 (0.011)*	0.948 (0.021)	0.915 (0.032)	0.983 (0.008)

a CNN: convolutional neural network.

b BiSRNN: bidirectional simple recurrent neural network.

c BiGRU: bidirectional gated recurrent unit.

**Figure 4.**
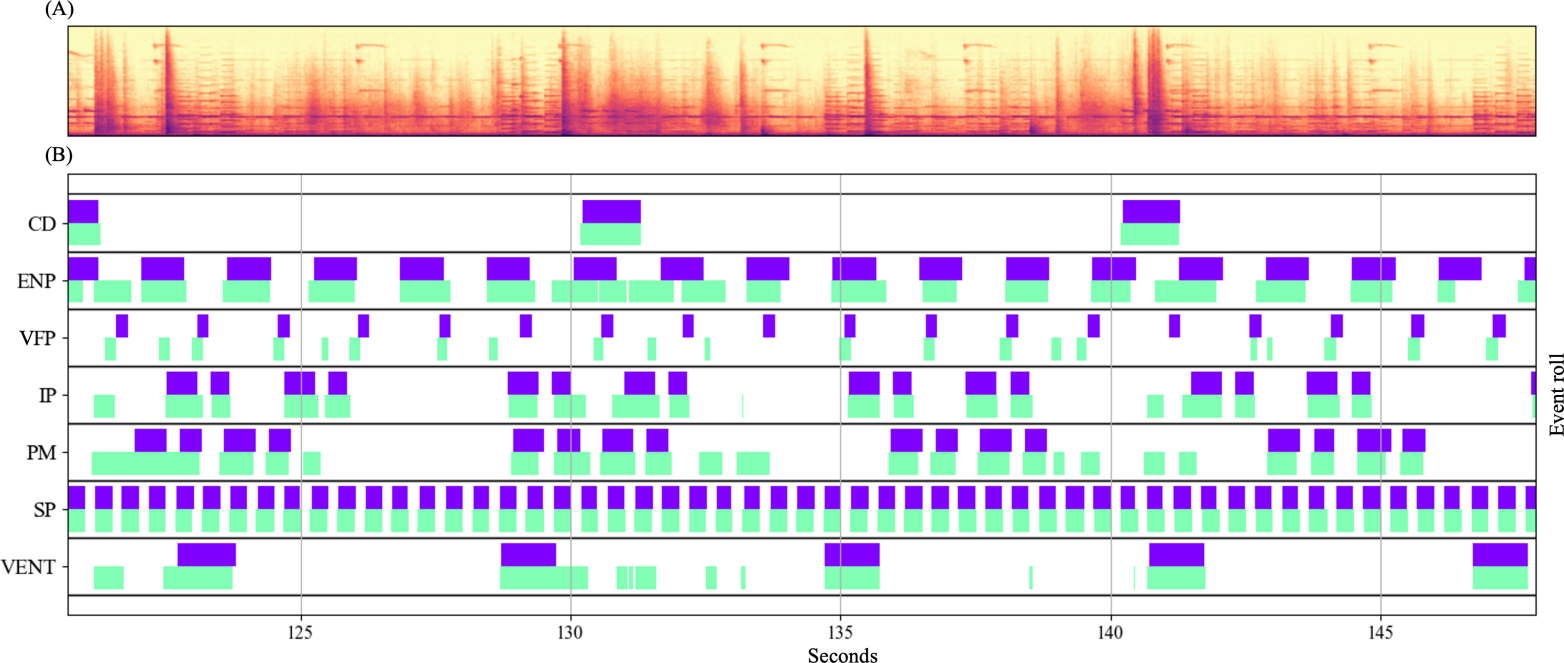
The reference and proposed model outputs of a convolutional neural network 4+bidirectional gated recurrent unit at a signal-to-noise ratio of 0 dB: (A) spectrogram of the simulation dataset and (B) event roll of the reference and proposed model outputs for the 7 devices. CD: chest drainage; ENP: enteral feeding pump; IP: infusion pump; PM: patient monitor; SP: syringe pump; VFP: venous foot pump; VENT: mechanical ventilator.

Table 6 shows the event-based class-wise metrics at an SNR of 0 dB. Only event-based metrics are reported here, as they reflect the temporal accuracy of onset and offset detection, which is essential for clinical alarm management, whereas class-wise segment-based metrics are less indicative of operational performance. Class-wise segment-based results are provided in Multimedia Appendix 1 [30] for reference. Event-based evaluation showed that CNN3+BiGRU and CNN4+BiGRU had a recall value of 0.990 or higher for all devices but VFP. In contrast, the ENP, PM, and ventilator had *F*_1_-scores of 0.900 or less due to their low precision. The reference standard was the correctly annotated label from the event roll that visualized the classification results, while the output was the model’s identified results. In the absence of sound, the device was detected visually (Figure 4).

**Table 6.** Event-based class-wise metrics at a signal-to-noise ratio of 0 dB across 5-fold cross-validation. The numbers in italics represent the optimal value for each class.

Metrics and class		*F*_1_-score, mean (SD)	Precision, mean (SD)	Recall, mean (SD)
CNN3^a^+BiSRNN^b^				
IP^c^		0.945 (0.016)	0.900 (0.028)	0.995 (0.001)
SP^d^		0.994 (0.004)	0.990 (0.008)	*0.998 (0.000)*
ENP^e^		0.903 (0.011)	0.831 (0.016)	0.990 (0.007)
VFP^f^		0.829 (0.013)	0.828 (0.036)	0.834 (0.050)
CD^g^		0.815 (0.023)	0.688 (0.034)	*1.000 (0.000)*
PM^h^		0.854 (0.020)	0.749 (0.031)	0.993 (0.003)
Vent^i^		0.752 (0.058)	0.606 (0.078)	*1.000 (0.000)*
CNN3+BiGRU^j^				
IP		0.982 (0.008)	0.967 (0.015)	0.997 (0.001)
SP		*0.998 (0.000)*	*1.000 (0.000)*	0.997 (0.001)
ENP		*0.969 (0.016)*	*0.939 (0.029)*	*1.000 (0.000)*
VFP		0.939 (0.037)	0.931 (0.030)	*0.951 (0.074)*
CD		0.904 (0.031)	0.826 (0.052)	*1.000 (0.000)*
PM		0.924 (0.025)	0.862 (0.043)	*0.997 (0.001)*
Vent		*0.834 (0.034)*	*0.717 (0.050)*	*1.000 (0.000)*
CNN4+BiGRU				
IP		*0.986 (0.005)*	*0.977 (0.009)*	0.995 (0.002)
SP		0.998 (0.000)	*1.000 (0.000)*	0.997 (0.001)
ENP		0.939 (0.033)	0.887 (0.058)	*1.000 (0.000)*
VFP		*0.943 (0.022)*	*0.947 (0.027)*	0.942 (0.054)
CD		0.870 (0.030)	0.770 (0.048)	*1.000 (0.000)*
PM		*0.954 (0.014)*	*0.916 (0.025)*	0.996 (0.002)
Vent		0.797 (0.054)	0.666 (0.080)	*1.000 (0.000)*
ALL-CNN4+BiGRU				
IP		0.942 (0.030)	0.896 (0.052)	0.995 (0.003)
SP		*0.998 (0.000)*	*1.000 (0.000)*	0.996 (0.001)
ENP		0.908 (0.056)	0.836 (0.090)	*1.000 (0.000)*
VFP		0.921 (0.047)	0.943 (0.046)	0.901 (0.057)
CD		*0.949 (0.018)*	*0.903 (0.033)*	*1.000 (0.000)*
PM		0.904 (0.020)	0.829 (0.032)	0.995 (0.003)
Vent		0.805 (0.051)	0.677 (0.074)	*1.000 (0.000)*

a CNN: convolutional neural network.

b BiSRNN: bidirectional simple recurrent neural network.

c IP: infusion pump.

d SP: syringe pump.

e ENP: enteral feeding pump.

f VFP: venous foot pump.

g CD: chest drainage.

h PM: patient monitor.

i Vent: mechanical ventilator.

j BiGRU: bidirectional gated recurrent unit.

## Discussion

### Principal Results

The proposed classifier was found to successfully detect the status of nearby medical devices. Although video-based alarm detection was also considered, it was not feasible to detect the monitors with cameras without the introduction of a blind spot. Therefore, SED was considered a more feasible approach than video recognition.

This study describes the construction of a classifier using a large amount of artificial noise data. This classifier was used to evaluate polyphonic alarm sounds among a simulation dataset of HWN. Because the classification target is a sine wave, we expected that the target could be achieved with simple neural networks. However, BiSRNN did not achieve the target performance, requiring the application of BiGRU. Both the frequency and time axes required advanced processing to recognize alarm sounds. In addition, a convolutional layer with 4 layers outperformed one with 3 layers. The *F*_1_-score of the classifier using BiGRU was 0.900 or higher, which was robust in the detection of polyphonic alarm sounds. Only VFP in each proposed classifier was unclear in the spectrogram due to VFP having the lowest sound pressure level of the 7 devices, thus resulting in low recall. In addition, masking of an impact sound over the entire frequency axis of the spectrogram would result in lower precision and overdetection.

### Clinical Application as a Notification System

The notification system should alert the hospital information system without missing any situations, whether alarms are sounding on a single device or on multiple devices simultaneously. Therefore, recall of class-wise metrics was considered the most important in evaluating clinical applications. In particular, recall for the ventilator, the most essential support device of the 7 evaluated, was 1.000.

CNN4+BiGRU had the highest *F*_1_-score in the event-based overall metrics, and the recall values of ENP, CD, and the ventilator were 1.000 each. Only VFP showed a recall value below 0.950, primarily due to its lower sound pressure level. In some cases, detection occurred slightly earlier than the actual onset, which reduced the measured recall. However, the ventilator demonstrated low precision with frequent overdetections, suggesting that it could not be evaluated clinically because it could cause alarm fatigue [35]. In contrast, because the *F*_1_-scores of ENP, VFP, and CD without external output were 0.900 or higher, the system was likely feasible to notify staff of the alarm status of medical devices that could not be connected to the hospital information system. Therefore, the proposed system demonstrated feasibility as an alarm sound detection system and can be further refined for clinical use.

### Integration Into Hospital Networks

#### Data Privacy Implications

The system processes only nonspeech alarm signals, ensuring that no patient-identifiable audio is stored or transmitted. When integrated into hospital networks, alarm classifications should be anonymized at the edge, transmitting only classification results in compliance with privacy regulations such as the General Data Protection Regulation and Japan’s Act on the Protection of Personal Information.

#### Real-Time Processing Feasibility

Our classifier was designed for lightweight deployment, with feature extraction and model inference feasible on edge devices. The processing latency is on the order of milliseconds per input window, enabling real-time alarm monitoring without delaying clinical response.

#### Regulatory Concerns

Integration of an alarm detection system into hospital infrastructure would require compliance with medical device standards, including ISO/IEC 60601-1-8 for alarm systems. Depending on the jurisdiction, such a system may be classified as a medical device software, requiring regulatory approval. Early consultation with regulatory authorities is recommended to ensure compliance and patient safety.

### Limitations

This study had several limitations. First, the study evaluated only a limited range of medical devices from a single hospital, potentially limiting generalizability. Second, differences in hospital architecture (eg, room layouts, wall materials, and ambient noise) may affect sound propagation and detection. Third, despite ISO/IEC 60601-1-8 regulations, variations in alarm sounds across manufacturers and models were not assessed. Finally, large-scale live recording and annotation remain impractical; future work should use synthetic datasets and validate performance across diverse environments and device types.

### Comparison With Prior Work

Several reports have examined the classification of alarm sounds using deep learning. Evaluation of single sounds of horns and bicycle bells found that the 5-layer deep neural network that applied an integrated judgment process had *F*_1_-scores of 0.99 or higher [4]. Because horns and similar devices do not create sine waves of digital sounds, they cannot be evaluated in the same way as alarm sounds of medical devices. A study of alarm sounds of medical devices in a neonatal intensive care unit found that most of the alarms had SNRs of 0 dB or higher [8]. That study, however, did not consider a classifier for polyphonic alarm sounds. A classifier using CRNN was found to be effective for polyphonic acoustic sounds [26,36].

### Conclusions

Missed medical device alarms can lead to serious adverse events. To mitigate such risks, we developed a deep learning–based classifier for detecting polyphonic alarm sounds in hospital environments.

Alarm sounds emitted by medical devices are regulated by ISO/IEC 60601-1-8. Because this standard defines different tones and patterns for each device and priority, SED is expected to identify the device and priority successfully. Thus, we considered SED appropriate to determine alarm status. Automatic identification of alarm sounds in hospital rooms would facilitate safer medical care.

In the simulation experiment, the polyphonic alarm sound classifier showed excellent performance, with an *F*_1_-score of 0.945 at an SNR of 0 dB. The proposed classifier demonstrated feasibility for clinical alarm sound detection and can be further optimized. When combined with network connectivity, this classifier could improve the notification of abnormal patient status detected by medical devices without requiring each device to be individually connected.

## Supplementary material

10.2196/35987 Multimedia Appendix 1 Segment-based class-wise metrics at a signal-to-noise ratio of 0 dB across 5-fold cross-validation.

## Abbreviations

BiGRU: bidirectional gated recurrent unit
BiSRNN: bidirectional simple recurrent neural network
CD: chest drainage
CNN: convolutional neural network
CRNN: convolutional recurrent neural network
ENP: enteral feeding pump
HWN: hospital ward noise
IEC: International Electrotechnical Commission
IP: infusion pump
ISO: International Organization for Standardization
MFB: mel filter bank
PM: patient monitor
RNN: recurrent neural network
SED: sound event detection
SNR: signal-to-noise ratio
SP: syringe pump
VFP: venous foot pump
