# Supplementary material for: Detection of Polyphonic Alarm Sounds From Medical Devices Using Frequency-Enhanced Deep Learning: Simulation Study
Source: JMIR Med Inform. 2025 Nov 12;13:e35987. doi: 10.2196/35987 (PMC12611226; doi:10.2196/35987)
Supplement: Multimedia Appendix 1 [file medinform-v13-e35987-s001.docx]

**Multimedia Appendix**

**Table S1**: Segment-based class-wise metrics at a signal-to-noise ratio of 0 dB across 5-fold cross-validation.

|  | Class | F | Precision | Recall |
| --- | --- | --- | --- | --- |
|  |  |  |  |  |
| **CNN3+BiSRNN** | IP | 0.946(0.008) | 0.962(0.007) | 0.931(0.012) |
|  | SP | 0.903(0.001) | 0.859(0.012) | 0.952(0.013) |
|  | ENP | 0.794(0.026) | 0.870(0.011) | 0.730(0.039) |
|  | VFP | 0.306(0.017) | 0.336(0.016) | 0.282(0.030) |
|  | CD | 0.922(0.021) | 0.877(0.035) | 0.972(0.004) |
|  | PM | 0.820(0.022) | 0.750(0.034) | 0.905(0.016) |
|  | VENT | 0.913(0.014) | 0.862(0.024) | 0.971(0.006) |
| **CNN3+BiGRU** | IP | 0.957(0.008) | 0.970(0.008) | 0.944(0.010) |
|  | SP | 0.903(0.002) | 0.845(0.010) | 0.969(0.015) |
|  | ENP | 0.903(0.016) | 0.908(0.024) | 0.898(0.010) |
|  | VFP | 0.373(0.047) | 0.395(0.037) | 0.356(0.055) |
|  | CD | 0.928(0.008) | 0.891(0.017) | 0.968(0.006) |
|  | PM | 0.877(0.018) | 0.842(0.029) | 0.917(0.006) |
|  | VENT | 0.915(0.023) | 0.859(0.038) | 0.981(0.003) |
| **CNN4+BiGRU** | IP | 0.962(0.003) | 0.969(0.007) | 0.955(0.003) |
|  | SP | 0.904(0.001) | 0.847(0.005) | 0.970(0.006) |
|  | ENP | 0.885(0.018) | 0.914(0.017) | 0.858(0.030) |
|  | VFP | 0.455(0.030) | 0.484(0.020) | 0.432(0.045) |
|  | CD | 0.928(0.007) | 0.899(0.013) | 0.960(0.004) |
|  | PM | 0.884(0.007) | 0.867(0.018) | 0.903(0.015) |
|  | VENT | 0.908(0.024) | 0.848(0.043) | 0.979(0.003) |
| **ALL-CNN4+BiGRU** | IP | 0.941(0.005) | 0.923(0.007) | 0.961(0.005) |
|  | SP | 0.904(0.003) | 0.849(0.008) | 0.967(0.012) |
|  | ENP | 0.881(0.021) | 0.877(0.034) | 0.886(0.028) |
|  | VFP | 0.430(0.064) | 0.475(0.056) | 0.393(0.069) |
|  | CD | 0.958(0.008) | 0.946(0.013) | 0.970(0.004) |
|  | PM | 0.856(0.013) | 0.799(0.016) | 0.921(0.014) |
|  | VENT | 0.921(0.019) | 0.867(0.032) | 0.982(0.003) |
